# Dendritic Spike Saturation of Endogenous Calcium Buffer and Induction of Postsynaptic Cerebellar LTP

**DOI:** 10.1371/journal.pone.0004011

**Published:** 2008-12-23

**Authors:** Marco Canepari, Kaspar E. Vogt

**Affiliations:** Division of Pharmacology and Neurobiology, Biozentrum–University of Basel, Basel, Switzerland; Tel Aviv University, Israel

## Abstract

The architecture of parallel fiber axons contacting cerebellar Purkinje neurons retains spatial information over long distances. Parallel fiber synapses can trigger local dendritic calcium spikes, but whether and how this calcium signal leads to plastic changes that decode the parallel fiber input organization is unknown. By combining voltage and calcium imaging, we show that calcium signals, elicited by parallel fiber stimulation and mediated by voltage-gated calcium channels, increase non-linearly during high-frequency bursts of electrically constant calcium spikes, because they locally and transiently saturate the endogenous buffer. We demonstrate that these non-linear calcium signals, independently of NMDA or metabotropic glutamate receptor activation, can induce parallel fiber long-term potentiation. Two-photon imaging in coronal slices revealed that calcium signals inducing long-term potentiation can be observed by stimulating either the parallel fiber or the ascending fiber pathway. We propose that local dendritic calcium spikes, evoked by synaptic potentials, provide a unique mechanism to spatially decode parallel fiber signals into cerebellar circuitry changes.

## Introduction

Neuronal dendrites can fire action potentials mediated by voltage-gated calcium channels (VGCC) that may be sufficient to induce long-term potentiation (LTP) of synaptic potentials [1], [2]. Calcium signals mediated by VGCCs differ from those mediated by ionotropic or metabotropic glutamate receptors. Whereas the latter signals co-localize with activated receptors, either by direct calcium influx or by secondary intracellular pathways, the extent of the former signals is determined by the spread of the dendritic spike. In the cerebellar Purkinje neuron (PN), calcium spikes can be elicited by parallel fiber (PF) stimulation and can be localized to small regions [3]. However, the function of calcium spikes elicited by neighboring presynaptic fibers is unknown. In particular, dendritic excitation has been associated with PF synaptic plasticity in relation to the climbing fibre (CF)-excitatory postsynaptic potential (EPSP) providing the calcium signal underlying coincident PF and CF detection and PF- long-term depression (LTD) [4], but the role of local PF-elicited calcium spikes in long-term synaptic plasticity is unexplored.

In this report we show that PF-elicited dendritic calcium spikes can induce postsynaptic PF-LTP in the mouse cerebellum. We demonstrate that this phenomenon is correlated with the ability of high-frequency calcium spike bursts to locally and transiently saturate the endogenous calcium buffer (ECB) leading to supra-linear summation of intracellular free calcium concentration changes (*Δ[Ca^2+^]_i_*). Since dendritic excitation depends on the spatiotemporal summation of synaptic inputs, the present results suggest that a major physiological role of PF-evoked dendritic calcium spikes is to functionally associate cerebellar granule cell axons synchronously targeting the same PN dendritic region.

## Results

### Dendritic calcium spikes and non-linear summation of calcium signals elicited by PF-EPSPs

**Figure 1:**
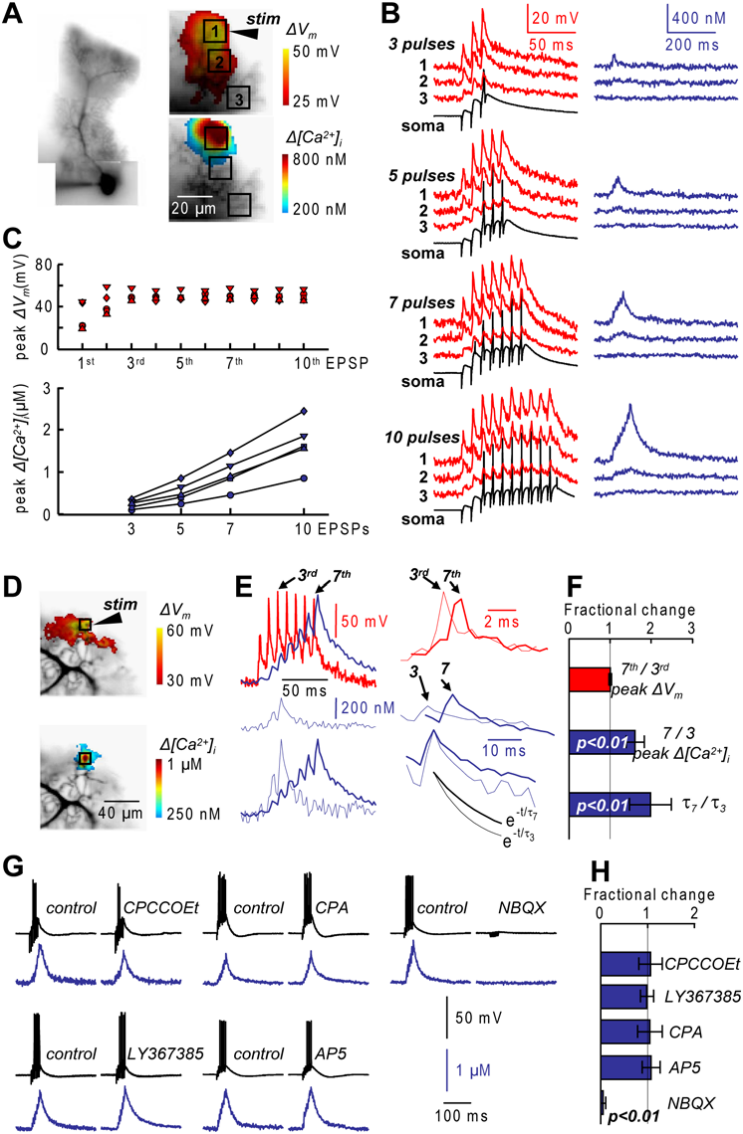
Dendritic *ΔV_m_* and *Δ[Ca^2+^]_i_* associated with PF-EPSPs. A. PN reconstruction (left) and recorded dendrites with three sample regions (8×8 pixels) and the position of the stimulating electrode; peak *ΔV_m_* and *Δ[Ca^2+^]_i_* signals following 10 PF-EPSPs at 100 Hz represented with color-coded scales. B. *ΔV_m_* and *Δ[Ca^2+^]_i_* signals associated with 3, 5, 7 and 10 PF-EPSPs in the three regions; somatic recordings reported; peak *ΔV_m_*>40 mV and detectable *Δ[Ca^2+^]_i_* signals in region 1 and in region 2 for the longer bursts. C. Peak *ΔV_m_* corresponding to 1–10 EPSPs and peak *Δ[Ca^2+^]_i_* following 3, 5, 7 and 10 EPSPs from 5 PNs; each symbol represents a different cell. D. Recorded dendrites with the 8×8 pixel region of maximal *Δ[Ca^2+^]_i_* signal; peak *ΔV_m_* and *Δ[Ca^2+^]_i_* signals following 7 PF-EPSPs at 100 Hz represented in color-code. E. (Left)-*ΔV_m_* and *Δ[Ca^2+^]_i_* recordings from the region in D following 7 PF-EPSPs; *Δ[Ca^2+^]_i_* recording following 3 PF-EPSPs reported above; normalized *Δ[Ca^2+^]_i_* recordings with 3 and 7 PF-EPSPs reported below. (Right)-Peak *ΔV_m_* and *Δ[Ca^2+^]_i_* signals corresponding to the 3^rd^ and 7^th^ EPSP; normalized peak *Δ[Ca^2+^]_i_* signals corresponding to the 3^rd^ and 7^th^ EPSP superimposed and single exponential decay fit (20 ms) reported below. F. Fractional changes (Mean±SD, N = 8 cells) of the 7^th^ peak *ΔV_m_* relative to the 3^rd^ peak, of the peak 7-EPSPs *Δ[Ca^2+^]_i_* relative to the peak 3-EPSPs *Δ[Ca^2+^]_i_* and of the 7-EPSPs *Δ[Ca^2+^]_i_* decay time constant (τ_7_) relative to the 3-EPSPs *Δ[Ca^2+^]_i_*; decay time constant (τ_3_). G. *Δ[Ca^2+^]_i_* recordings following 7 PF-EPSPs at 100 Hz before (control) and 10–15 minutes after addition of 100 µM CPCCOEt, 100 µM LY367385, 30 µM CPA, 100 µM AP5 or 10 µM NBQX as indicated; EPSPs and *Δ[Ca^2+^]_i_* signals blocked by NBQX. H. Fractional changes (Mean±SD) of the peak *Δ[Ca^2+^]_i_* signal after addition of a drug; CPCCOEt, LY367385, CPA or AP5: paired t-test, p>0.1, N = 7; of NBQX: paired t-test, p<0.01, N = 6.

Dendritic membrane potential and calcium signals were optically investigated in mouse cerebellar sagittal slices. We measured changes in dendritic membrane potential (*ΔV_m_*) and in intracellular free calcium concentration (*Δ[Ca^2+^]_i_*) signals in PNs loaded with the voltage sensitive dye JPW-1114 and 1 mM of the low-affinity calcium indicator Fura-FF as previously described [5]. High frequency trains of PF-EPSPs can elicit calcium spikes that mediate *Δ[Ca^2+^]_i_* which depend on the number of stimuli [6]. In the example of Figure 1A, PF stimulation in the vicinity of a dendritic branch with 3, 5, 7 and 10 pulses at 100 Hz elicited local *ΔV_m_* and *Δ[Ca^2+^]_i_* signals which declined sharply with the distance from the site of stimulation (Figure 1B). In the burst of 10 EPSPs, the somatic response facilitated from ∼5 mV to ∼20 mV after the third pulse with episodes of somatic action potentials. The amplitude of the somatic EPSPs was comparable to the size of the *ΔV_m_* signal measured in the majority of the dendritic field. However, in the area where *Δ[Ca^2+^]_i_* signals were detected, the peak *ΔV_m_* signal exceeded 40 mV (Figure 1A). This value compares with the size of dendritic calcium spikes measured with electrode dendritic recordings [7], [3]. Notably, the size of the dendritic calcium spike associated with the 3^rd^–10^th^ EPSP did not change, but the *Δ[Ca^2+^]_i_* increased supra-linearly with the number of events. This result was observed in all 5 PNs tested for 3–10 PF EPSPs (Figure 1C).

The supra-linear *Δ[Ca^2+^]_i_* signal described above was due to two factors as shown in the experiment in Figure 1D where *Δ[Ca^2+^]_i_* signals were analyzed for 3 and 7 EPSPs. First, the contribution to the *Δ[Ca^2+^]_i_* signal of each calcium spike increased during the burst. Second, the fast component of the decay time constant (τ) of the *Δ[Ca^2+^]_i_* signals decreased with the number of EPSPs, enhancing the summation of *Δ[Ca^2+^]_i_* contributions from individual spikes. In Figure 1E the *Δ[Ca^2+^]_i_* signals associated with the 3^rd^ and 7^th^ EPSPs are superimposed and normalized to their maxima. Single exponential functions are fitted to the initial 20 ms decay of the two *Δ[Ca^2+^]_i_* signals. In contrast to the marked change in the *Δ[Ca^2+^]_i_* signal, the difference in the peak *ΔV_m_* signal associated with the 3^rd^ and 7^th^ EPSPs was minimal. As shown in Figure 1F, in 8 cells with a peak *Δ[Ca^2+^]_i_* signal of 0.7–1.5 µM after 7 PF-EPSPs, the *Δ[Ca^2+^]_i_* contribution of the 7^th^ EPSP spike, relative to the 3^rd^, was higher (ratio: 1.66±0.22; paired t-test p<0.01) and the decay time constant of the *Δ[Ca^2+^]_i_* signal after 7 EPSPs, relative to the *Δ[Ca^2+^]_i_* signal after 3 EPSPs, slower (ratio: 1.98±0.51; paired t-test p<0.01). However, the peak *ΔV_m_* associated with the 7^th^ EPSP, relative to the peak *ΔV_m_* associated with the 3^rd^ EPSP, was not different (ratio: 1.00±0.03, paired t-test p>0.2), indicating no change in the calcium spikes during the burst.

The peak *Δ[Ca^2+^]_i_* signal followed the calcium spike by 2–4 ms, suggesting calcium spike influx independent of mGluR1-mediated signals or of influx via NMDA receptors, expressed in mature PNs [8]. *Δ[Ca^2+^]_i_* signals following 7 PF-EPSPs were not significantly affected by the addition of the mGluR1 antagonists CPCCOEt (100 µM) and LY367385 (100 µM), by the blocker of endoplasmic reticulum calcium-ATPase Cyclopiazonic acid (CPA, 30 µM) or by blocking NMDA receptors with AP5 (100 µM) (Figure 1G). As shown in Figure 1H, The ratios between *Δ[Ca^2+^]_i_* signals before and (10–15 minutes) after addition of these drugs were 1.06±0.25, 0.98±0.14, 1.04±0.26 and 1.07±0.19 respectively (N = 7, paired t-test p>0.1 in all three cases). The *Δ[Ca^2+^]_i_* signal was blocked by suppressing depolarization with the AMPA receptor antagonist NBQX (10 µM) (fractional *Δ[Ca^2+^]_i_* change: 0.05±0.05, N = 6, paired t-test p<0.01). As AMPA receptors do not contribute to dendritic calcium signals in PNs [6], we conclude that fast *Δ[Ca^2+^]_i_* signals associated with PF-stimulation spikes were mediated by VGCCs.

**Figure 2:**
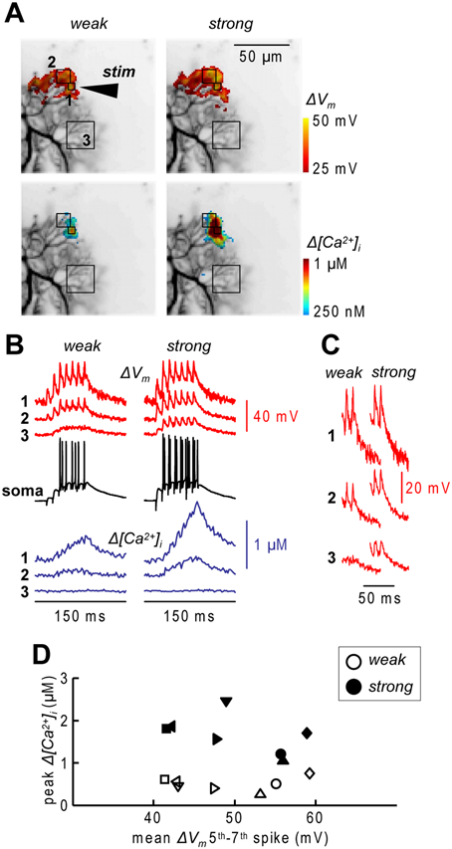
Dendritic *ΔV_m_* and *Δ[Ca^2+^]_i_* while increasing the number of stimulated PFs. A. Recorded dendrites with three sample regions (1–3). Peak *ΔV_m_* and *Δ[Ca^2+^]_i_* following 7 PF-EPSPs at 100 delivered by the electrode “stim” at a “weak” and “strong” stimulation intensities represented in color-code. B. *ΔV_m_* and *Δ[Ca^2+^]_i_* recordings from the regions 1–3 following PF stimulation at the two stimulation intensities; somatic recordings also reported. C. *ΔV_m_* corresponding to the last two spikes following weak and strong PF stimulation. D. Scatter plot of peak *Δ[Ca^2+^]_i_* against peak *ΔV_m_* averaged over the last three spikes at two stimulation intensities from 7 cells; empty symbols: weak stimulation; filled symbols: strong stimulation; each symbol represents a different cell.

The size of the *Δ[Ca^2+^]_i_* transients increases with the number of stimulated PF terminals and therefore with the excited dendritic area [6]. Figure 2A shows one example of a PN where *ΔV_m_* and *Δ[Ca^2+^]_i_* signals were measured following PF stimulation at two different intensities. The area of dendritic excitation and of the observable *Δ[Ca^2+^]_i_* signal enlarged with the stronger stimulation. In the region excited by the weaker stimulation, the peak *Δ[Ca^2+^]_i_* increased with the stronger stimulation (Figure 2B), but the peaks of *ΔV_m_* corresponding to the last spikes did not change (Figure 2C). The same result was observed in 7 cells tested with two stimulation intensities, as shown in the scatter plot of Figure 2D.

This first set of experiments shows that the non-linear *Δ[Ca^2+^]_i_* increase associated with PF-evoked dendritic spikes is independent of the electrical amplitude of the calcium spike.

### Dendritic calcium spikes and calcium signals elicited by CF-EPSPs

Dendritic calcium spikes can be also elicited by CF-EPSPs [9], but they are spread over a large area of the dendritic tree. In another set of experiments we examined whether and how supra-linear calcium signals can occur following CF stimulation.

**Figure 3:**
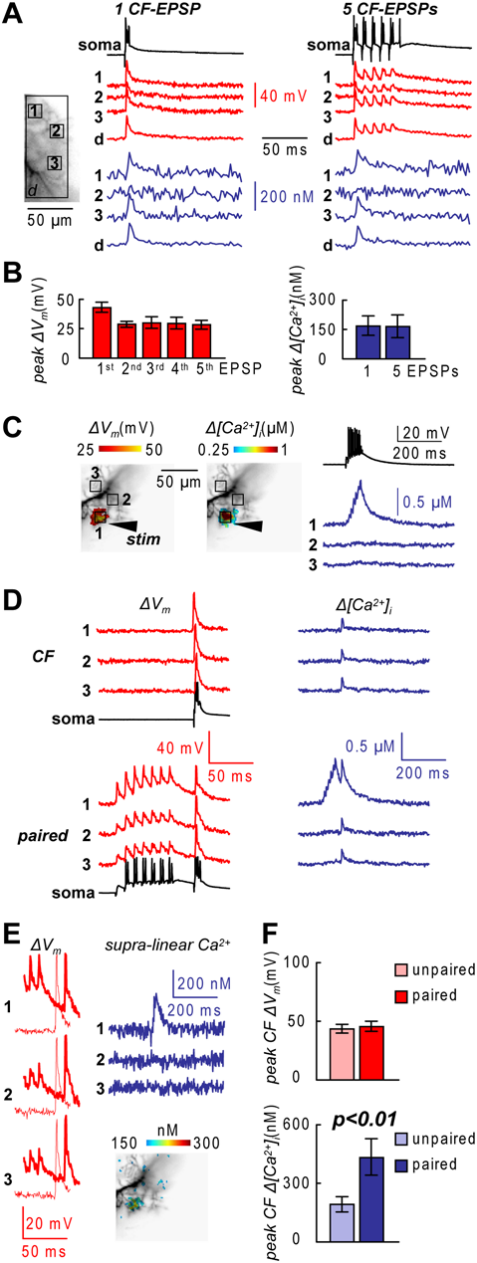
Dendritic *ΔV_m_* and *Δ[Ca^2+^]_i_* associated with CF-EPSPs. A. (Left) Recorded dendrites with three sample regions (1–3) and the whole dendritic regions (*d*) indicated. (Right)-*ΔV_m_* and *Δ[Ca^2+^]_i_* associated with 1 CF-EPSP or 5 CF-EPSPs at 100 Hz in the regions 1,2,3 and *d*; somatic recordings reported. B. Mean±SD of the peak *ΔV_m_* associated with 5 CF-EPSPs at 100 Hz and of the peak *Δ[Ca^2+^]_i_* associated with 1 CF-EPSP and 5 CF-EPSPs at 100 Hz from 4 PNs over the imaged dendritic area. C. (Left)-Recorded dendrites with three sample regions (1–3); peak *ΔV_m_* and *Δ[Ca^2+^]_i_* following 7 PF-EPSPs at 100 Hz delivered by “stim” represented in color-code. (Right)-Corresponding *Δ[Ca^2+^]_i_* recordings from the regions 1–3; somatic recording reported. D. *ΔV_m_* and *Δ[Ca^2+^]_i_* in the regions 1–3 following 1 CF EPSP unpaired (CF) and paired to 7 PF-EPSPs with a delay of 90 ms; paired CF-mediated *Δ[Ca^2+^]_i_* signal larger in the region excited by the PF-EPSPs. E. (Left)–*ΔV_m_* associated with the CF-EPSP in unpaired and paired conditions; no change in the CF *ΔV_m_* signal. (Right)–Supra-linear *Δ[Ca^2+^]_i_* from the difference between *Δ[Ca^2+^]_i_* associated with the pairing protocol and *Δ[Ca^2+^]_i_* associated with the unpaired PF-EPSPs and CF-EPSP; color-coded image is the supra-linear *Δ[Ca^2+^]_i_*. F. Mean±SD of the peak CF *ΔV_m_* and *Δ[Ca^2+^]_i_* associated 1 CF-EPSP unpaired or paired to 7 PF-EPSPs from 6 PNs; two populations t-tests: *ΔV_m_* signals, p>0.1; *Δ[Ca^2+^]_i_* signals, p<0.01.

CF-EPSP bursts are dominated by short-term depression due to presynaptic depletion [10], [11]. In the experiment shown in Figure 3A, *ΔV_m_* and *Δ[Ca^2+^]_i_* signals following either 1 CF-EPSP or 5 CF-EPSPs at 100 Hz were measured. Although CF activation occurs at low frequencies, it was important to perform this test to compare CF-mediated excitation with PF-mediated calcium spikes and because CF high frequency stimulation was used in another study [12]. The peak *ΔV_m_* signals corresponding to the 2^nd^–5^th^ EPSP were smaller compared to the first one. In most dendritic regions, the first EPSP of the train evoked a calcium transient, but nowhere a *Δ[Ca^2+^]_i_* signal associated with the 2^nd^–5^th^ EPSP was observed. In 4 cells, we measured *ΔV_m_* and *Δ[Ca^2+^]_i_* signals over the whole imaged dendritic area. The peak *ΔV_m_* signals of the 1^st^, 2^nd^, 3^rd^, 4^th^, and 5^th^ EPSP were 43.0±4.2 mV, 29.0±2.4 mV, 30.0±5.0 mV, 29.3.0±5.0 mV and 28.5±3.7 mV respectively, whereas the *Δ[Ca^2+^]_i_* signal following 1 CF-EPSPs and 5-CF EPSPs were unaltered (168±50 nM and 165±50 nM respectively, Figure 3B). The size of this *Δ[Ca^2+^]_i_* signal was in the range of what estimated in a different quantitative study [13]. However, it must be pointed out that our estimate could be biased by our approximate estimation of *Δ[Ca^2+^]_i_* (see Materials and Methods) and by the assumed dissociation constant of Fura-FF (*K_d_* = 10 µM) derived from the literature [14]. In summary, CF-EPSP high-frequency bursts are associated with small dendritic *Δ[Ca^2+^]_i_* signals because only the first EPSP of a train can evoke a calcium transient.

Although CF-mediated *Δ[Ca^2+^]_i_* signals are small, when paired with a short delay after PF-EPSPs bursts, CF-EPSPs are associated with a supra-linear dendritic *Δ[Ca^2+^]_i_* signal, independent of the activation of mGluR1s and calcium release from stores [12]. Therefore, we tested whether the *ΔV_m_* signal associated with the CF-EPSP was changed by the pairing protocol. Figure 3C shows one experiment in which *ΔV_m_* and *Δ[Ca^2+^]_i_* signals were measured for a pairing protocol with one CF-EPSP delayed by 90 ms from the beginning of a 7 PF-EPSPs burst. In the region of PF-evoked dendritic calcium spikes, the *Δ[Ca^2+^]_i_* signal associated with the CF-EPSP increased during the pairing protocol (Figure 3D). The site of the supra-linear *Δ[Ca^2+^]_i_* signal (Figure 3E), obtained as the difference between the *Δ[Ca^2+^]_i_* signals during paired and unpaired stimulation, co-localized with the region of the PF-evoked dendritic spikes (Figure 3C). However, the dendritic depolarization associated with the CF-EPSP did not change following the pairing protocol (Figure 3E), indicating that the previous PF-EPSPs burst did not affect the CF-evoked calcium spike. In 6 cells (Figure 3F), in the region excited by the PF-EPSPs burst, the paired peak CF-associated *Δ[Ca^2+^]_i_*, obtained by subtracting the PF- *Δ[Ca^2+^]_i_* from the paired *Δ[Ca^2+^]_i_*, was 433±33 nM, higher (p<0.01, two-sample t-test) than the unpaired peak CF *Δ[Ca^2+^]_i_* (192±50 nM). In contrast, the paired peak CF *ΔV_m_* (43.5±7.7 mV) did not change from the unpaired CF *ΔV_m_* (45.7±8.3 mV, p>0.2 two-sample t-test). Therefore, the dendritic supra-linear calcium signal, associated with paired PF and CF stimulation, is independent of the *ΔV_m_* peak of the CF-evoked calcium spike.

### Supra-linear *Δ[Ca^2+^]_i_* signals associated with calcium spikes are due to local saturation of the endogenous calcium buffer

The evidence that progressively larger spike-*Δ[Ca^2+^]_i_* components do not correlate with changes in the electrical amplitude of dendritic calcium spikes excludes the possibility of increasing calcium influx and indicates a saturation of calcium binding proteins forming the ECB. To directly estimate the amount of calcium influx, we sequentially patched PNs first with Fura-FF to measure *Δ[Ca^2+^]_i_* signals and later with 10 mM of the high-affinity calcium indicator Bis-Fura-2 (BF2). We estimated the amount of calcium bound to the non-saturated high-affinity calcium indicator BF2 as described in the Materials and Methods.

It must be pointed out that BF2 injection blocks calcium dependent processes preventing the test of putative calcium-dependent modulations on calcium influx. However, calcium down-regulates calcium spikes by activating calcium-gated potassium channels [3], which are coupled to P/Q type calcium channels [15] and this mechanism would only decrease calcium influx.

**Figure 4:**
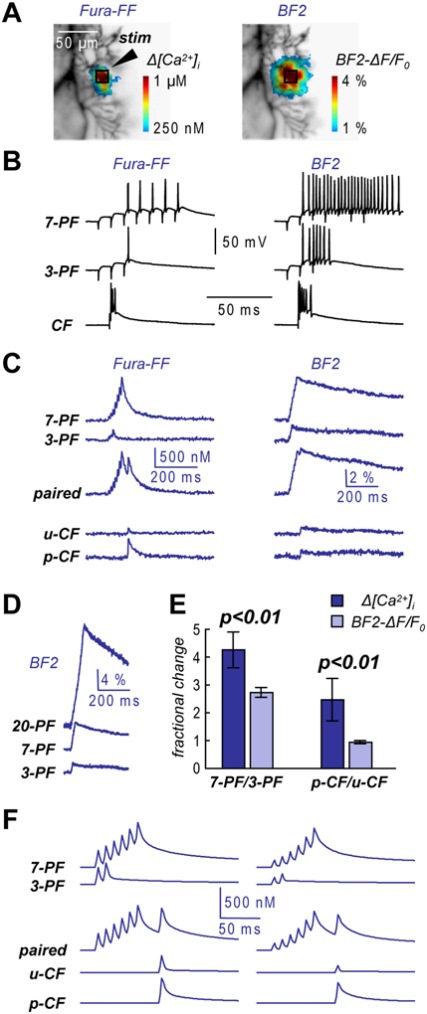
Dendritic calcium influx measured with BF2. A. Recorded dendrites with the color-coded fluorescence signals following 7 PF-EPSPs at 100 Hz from 1 mM Fura-FF and 10 mM BF2; the position of the stimulating electrode “stim” and the 8×8 pixels region of maximal *Δ[Ca^2+^]_i_* indicated; the area of the BF2 signal wider. B. Somatic recordings following 7 PF-EPSPs, 3 PF-EPSPs and 1 CF-EPSP 15 minutes after whole-cell with 1 mM Fura-FF (left) and 30 minutes after whole-cell with 10 mM BF2 (right); increased somatic excitability with BF2. C. *Δ[Ca^2+^]_i_* (left) and *BF2-ΔF/F_0_* (right) following 7 PF-EPSPs, 3 PF-EPSPs, a PF-CF pairing (*paired*), 1 unpaired CF-EPSP (*u-CF*); the *Δ[Ca^2+^]_i_* corresponding the paired CF-EPSP (*p-CF*) calculated by subtracting the 7-PF signal from the *paired* signal also reported. D. *BF2-ΔF/F_0_* following 20 PF-EPSPs, 7 PF-EPSPs and 3 PF-EPSPs indicating no saturation of the indicator. E. Fractional changes (Mean±SD, N = 5 cells) of the peak *Δ[Ca^2+^]_i_* and of the BF2-*ΔF/F_0_* of the 7 PF-EPSPs signals relative to the 3 PF-EPSPs signal and of the *p-CF* signals relative to the *u-CF* signals; paired t-test on the two fractional changes: p<0.01. F. Computer simulations of *Δ[Ca^2+^]_i_* according to the model described in the Materials and Methods; (Left): CB concentration 100 µM, fast CB binding site *K_on_* 8.53·10^7^ M^−1^s^−1^; (Right): CB concentration 100 µM, Fast CB binding site *K_on_* 34.12·10^7^ M^−1^s^−1^; note the better agreement of the right traces with experimental scenarios.

In the cell of Figure 4A the region where a change of fluorescence (either a *Δ[Ca^2+^]_i_* or *BF2-ΔF/F_0_* signal) was detected following 7 PF-EPSPs was wider in the presence of BF2, a phenomenon possibly due to the diffusion of the bound-indicator and to a larger firing region in conditions where activation of the calcium-activated BK channels is prevented [3]. In addition, the block of calcium-activated potassium channels (in particular SK channels) increased somatic firing [16] during both PF and CF stimulation (Figure 4B). Taking into account these effects due to BF2 injection, we compared *Δ[Ca^2+^]_i_* signals and *BF2-ΔF/F_0_* signal as shown in Figure 4C. The 7 PF-EPSPs relative to 3 PF-EPSPs *BF2-ΔF/F_0_* was smaller than the corresponding *Δ[Ca^2+^]_i_* signals ratio and no paired CF-associated supra-linear *BF2-ΔF/F_0_* signal was detected. These results were not due to BF2 saturation, since 20-PF-EPSPs induced a BF2-ΔF/F_0_ ∼3.5 times larger than that associated with 7 PF-EPSPs (Figure 4D). In N = 5 cells (Figure 4E), the 7 PF-EPSPs relative to 3 PF-EPSPs *BF2-ΔF/F_0_* (2.48±0.76) was smaller than the corresponding control *Δ[Ca^2+^]_i_* signals ratio (4.26±0.64, paired t-test: p<0.01). The paired CF-*BF2-ΔF/F_0_* signal was almost identical to the unpaired CF-*BF2-ΔF/F_0_* signal (ratio: 0.96±0.06) compared to the larger paired CF- *Δ[Ca^2+^]_i_* signal (ratio: 2.73±0.17, paired t-test: p<0.01). We concluded that in conditions of enhanced excitation by the block of calcium-activated potassium channels, calcium influx is constant for consecutive calcium spikes and their individual contributions to *BF2-ΔF/F_0_* summate linearly.

To finally confirm that ECB saturation can occur during dendritic calcium bursts, we ran computer simulations using the single compartment model of dendritic calcium dynamics in PN dendrites described by Schmidt et al. [13]. The model predicts the time course of free calcium concentration in the presence of the ECBs calbindin D_28k_ (CB) and parvalbumin (PV). We began the analysis by using the same set of conditions of Schmidt et al. [13] and, from this starting point, we modified the parameters according to our different conditions (different calcium indicator, different Mg-ATP concentration and different temperature) as described in detail in the Materials and Methods. In particular, we referred to the published values for the kinetics of CB [17] and PV [18] and doubled the on-rate (*K_on_*) and the dissociation constant (*K_d_*) to account for the different temperature (32-33°C instead of room temperature). As shown by Schmidt et al. [13], CB affects the peak amplitude and the fast components of *Δ[Ca^2+^]_i_* signals whereas PV does not. Thus, in order to obtain a *Δ[Ca^2+^]_i_* signal amplitude of ∼300 nM, for a single calcium spike, we set the CB concentration to 100 µM, a value that is larger than that used by Schmidt et al. [13], but still smaller than that estimated in another study where dendritic ECB saturation was reported [19]. Assuming that the first PF-EPSP of a train of 7 EPSPs does not elicit a calcium spike, we simulated the experimental scenarios of Figure 4 with 2 and 6 calcium spikes at 100 Hz. We also set the occurrence of the CF-EPSP 80 ms after the first calcium spike. The results of this first simulation are reported in Figure 4F (left traces). Dendritic ECB saturation, in particular of CB, is predicted by the model. Interestingly, both *Δ[Ca^2+^]_i_* amplitude and its degree of non-linearity during consecutive spikes depend not only on the CB concentration, but also on its kinetics and affinity. Figure 4F (right traces) shows the result of a second simulation in which the on-rate and the affinity of the faster binding site of CB were set to a value 4 times larger than that in the previous simulation. In this condition the first *Δ[Ca^2+^]_i_* amplitude was 165 nM and the supralinear increase of the *Δ[Ca^2+^]_i_* signal was more prominent and closer to the experimental observation. This qualitative result suggests that faster ECB other than CB might play a significant role in the phenomenon described here.

### PF-evoked dendritic spikes induce PF long-term synaptic plasticity

**Figure 5:**
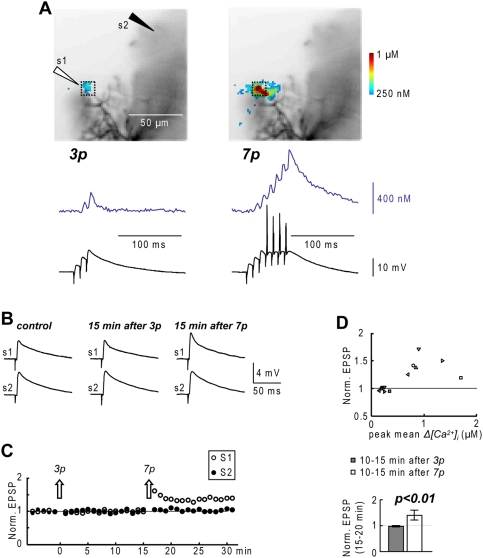
Repetitive PF-EPSPs bursts induce PF-LTP. A. (Top)–Recorded dendrites with the position of two stimulating electrodes (s1 and s2) and the mean *Δ[Ca^2+^]_i_* peak associated with repetitive 3-EPSPs bursts (3p) and 7-EPSPs bursts (7p) delivered by s1 represented in color-code. (Bottom)- *Δ[Ca^2+^]_i_* recordings in the maximal 8×8 pixels region and somatic recordings associated with 3p (left) and 7p (right) protocols. B. Average of 20 trials following s1 and s2 stimulation in control, 10-15 minutes after the 3p protocol and 10–15 minutes after 7p protocol. C. Time course of EPSP amplitudes normalized to control EPSP amplitudes (20 EPSPs in 5 minutes) evoked by s1 and s2; each point is the average of 4 EPSPs in one minute; the arrows indicate the time of the 3p and 7p protocols (s1). D. (Top)-Scatter plot of mean normalized EPSP 10–15 after a 3p protocol and after a 7p protocol against peak *Δ[Ca^2+^]_i_*; each symbol is a different cell. (Bottom)-Mean±SD of normalized EPSP 10–15 minutes after a 3p protocol and 10–15 after a 7p protocol; paired t-test: p<0.01.

The transient saturation of ECB leads to a fast and relatively large *Δ[Ca^2+^]_i_* signal that may induce synaptic plasticity. To test this hypothesis, we explored the effect of repetitive bursting activity on the amplitude of the PF-EPSPs, recorded every 15 s (4 EPSPs every minute, 0.067 Hz). In the experiment of Figure 5, we tested first the effect of repeating 3 PF-EPSPs for 60 times at 1 Hz (typically *Δ[Ca^2+^]_i_* below ECB saturation) and later the effect of repeating 7 PF-EPSPs (*Δ[Ca^2+^]_i_* above ECB saturation). In a 8×8 pixels region, 3 EPSPs were associated with 2 calcium spikes and a *Δ[Ca^2+^]_i_* signal of ∼200 nM whereas 7 EPSPs were associated with 6 calcium spikes and a *Δ[Ca^2+^]_i_* signal of ∼900 nM (Figure 5A). Repetitive application of 3 EPSPs didn’t cause any change in the EPSP amplitude tested up to 15 minutes later, whereas repetitive application of 7 EPSPs caused a robust LTP (Figure 5B and Figure 5C). The scatter plot and the bar diagram in Figure 5D show the fractional change of the EPSP amplitude 10-15 minutes following repetitive stimulation with 3 and 7 EPSPs (summary results from 6 cells). 3 EPSPs which evoked peak *Δ[Ca^2+^]_i_* signals <300 nM never affected the EPSP amplitude whereas 7 EPSPs associated with peak *Δ[Ca^2+^]_i_* signals >600 nM consistently caused LTP.

To better characterize this phenomenon, we used a protocol (conditioning protocol) of 7 EPSPs at 100 Hz (EPSP burst) repeated 60 times at 1 Hz. By adjusting the stimulation intensity, we explored the effect of *Δ[Ca^2+^]_i_* signals of different size. To standardize the analysis, we used the 8×8 pixel region of maximal *Δ[Ca^2+^]_i_* size to correlate *Δ[Ca^2+^]_i_* signals ranging from 0.1 µM (the minimal detectable *Δ[Ca^2+^]_i_*) up to 4 µM, with the change in amplitude of the PF-EPSPs.

**Figure 6:**
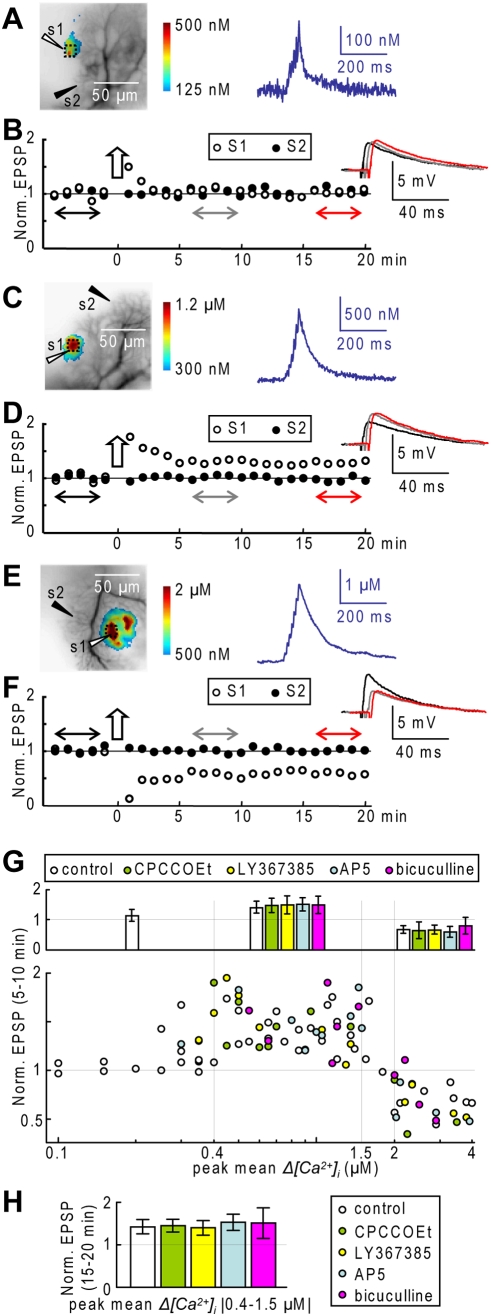
PF-evoked PF long term plasticity depends on the *Δ[Ca^2+^]_i_* peak. A. (Left)–Recorded dendrites with the position of stimulating electrodes s1 and s2 and the *Δ[Ca^2+^]_i_* peak following a conditioning protocol delivered by s1 in color-code. (Right)- *Δ[Ca^2+^]_i_* signal of ∼250 nM in its maximal 8×8 pixels region (average of 30 trials). B. Time course of the normalized EPSP amplitudes (20 EPSPs in 5 minutes) evoked by s1 and s2; each point is the average of 4 EPSPs; the arrow indicates the s1 conditioning protocol; traces are averages of 20 EPSPs before, 5–10 minutes after and 15–20 minutes after the conditioning protocol. C and D. Same as A and B in another cell with mean *Δ[Ca^2+^]_i_* ∼1 µM. E and F. Same as A and B in another cell with mean *Δ[Ca^2+^]_i_* ∼2 µM. G. (Bottom)-Semi-logarithmic scatter plot of mean normalized EPSP 5–10 after the conditioning protocol against peak *Δ[Ca^2+^]_i_* during the conditioning protocol; values in control condition: 35 cells and 45 dendritic locations; CPCCOEt (100 µM): 12 dendritic locations; LY367385 (100 µM): 12 dendritic locations; AP5 (100 µM): 13 dendritic locations; bicuculline (20 µM): 10 dendritic locations. (Top)-Mean±SD of normalized EPSP 5–10 minutes after conditioning protocol in the ranges of 0.1–0.4 µM *Δ[Ca^2+^]_i_*, of 0.4–1.5 µM *Δ[Ca^2+^]_i_* and of 2–4 µM *Δ[Ca^2+^]_i_*. H. Mean±SD of normalized EPSP 15–20 minutes after conditioning protocol in the range of 0.4–1.5 µM *Δ[Ca^2+^]_i_*.

Figure 6 shows three representative experiments with conditioning protocols adjusted to obtain peak *Δ[Ca^2+^]_i_* signals of ∼0.2 µM, ∼1 µM and ∼2 µM. In the first example (Figure 6A and Figure 6B), the amplitude of the PF-EPSPs, after a transient post-tetanic potentiation, returned to the initial EPSP amplitude after ∼2 minutes. In the second example (Figure 6C and Figure 6D), the transient post-tetanic potentiation was followed by an LTP of the PF-EPSPs lasting for more than 20 minutes. In the last example (Figure 6E and Figure 6F) the conditioning protocol induced a long-term depression (LTD) of the PF-EPSP, lasting for more than 20 minutes. The scatter plot of Figure 6G summarizes the changes in PF-EPSP amplitudes as a function of the peak *Δ[Ca^2+^]_i_* after 5–10 minutes obtained from 34 cells for a total of N = 45 dendritic locations tested. The graph of Figure 6H quantifies the dependence of plasticity from the dendritic *Δ[Ca^2+^]_i_* signal. In the range of 0.1–0.4 µM the PF-EPSP was occasionally potentiated but in the majority of the cases it was not affected by the conditioning protocol (changes in EPSP amplitude after 5–10 minutes and after 15–20 minutes: 1.14±0.20, N = 14 and 1.15±0.25, N = 6, respectively). In contrast, in the range of 0.4–1.5 µM, the PF-EPSP was consistently potentiated by the conditioning protocol; the change in EPSP amplitude after 5–10 minutes and after 15–20 minutes was 1.39±0.20 (N = 20) and 1.42±0.17 (N = 10), respectively. Finally, in the range of 2–4 µM the conditioning protocol consistently induced LTD (change in the EPSP amplitude after 5–10 minutes and after 15–20 minutes: 0.67±0.13, N = 8 and 0.58±0.07, N = 4, respectively).

The profile of long-term plasticity as a function of the *Δ[Ca^2+^]_i_* signal confirms the results reported in another study [20]. This profile was maintained in the presence of the mGluR1 antagonists CPCCOEt (100 µM, 7 cells and N = 12 dendritic locations) or LY367385 (100 µM, 8 cells and N = 12 dendritic locations) and of the NMDA receptor antagonist AP5 (100 µM, 10 cells and N = 13 dendritic locations). In the presence of CPCCOEt, LY367385 and NMDA respectively, the fractional change in EPSP amplitude in the range of 0.4–1.5 µM was 1.47±0.24 (N = 8), 1.49±0.30 (N = 7) and 1.51±0.22 (N = 9) after 5–10 minutes and 1.45±0.15 (N = 4), 1.40±0.17 (N = 4) and 1.53±0.19 (N = 4) after 20 minutes, whereas in the range of 2–4 µM the fractional change after 5–10 minutes was 0.64±0.28 (N = 4), 0.65±0.15 (N = 4) and 0.60±0.18 (N = 4). Stimulation in control conditions could involve long-term plasticity mediated by inhibitory synaptic potentials. To test this hypothesis in 7 cells (N = 10 dendritic locations) we did experiments in the presence of the GABA_A_ receptor antagonist bicuculline (20 µM). The fractional change in EPSP amplitude in the range of 0.4–1.5 µM was 1.50±0.29 (N = 6) after 5–10 minutes and 1.51±0.36 (N = 4) after 20 minutes, whereas in the range of 2–4 µM it was 0.79±0.28 (N = 4) after 5–10 minutes, indicating no effect of bicuculline.

**Figure 7:**
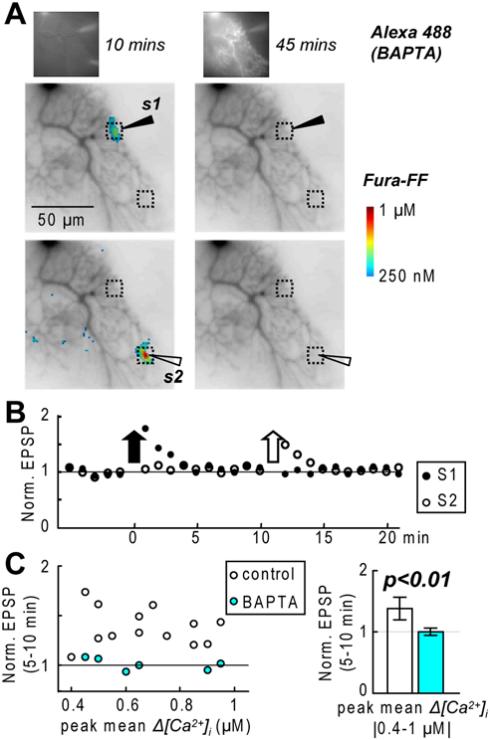
Postsynaptic induction of PF-LTP. A. (Insets)-Alexa-488 fluorescence from a PN 10 minutes (left) and 45 minutes (right) after whole cell; the pipette filled from the tip with clear solution and from the back with 25 mM BAPTA and 100 µM Alexa-488. Corresponding Fura-FF fluorescence from the same PN; *Δ[Ca^2+^]_i_* peak following 7 stimuli delivered by s1 (top) and s2 (bottom) represented in color code. B. Time course of normalized EPSP amplitudes evoked by s1 and s2 (20 EPSPs in 5 minutes); the arrows indicate the time of the conditioning protocols. C. (Left)-Scatter plot of the mean normalized EPSP 5–10 after conditioning protocol against the peak *Δ[Ca^2+^]_i_* associated with a conditioning protocol; control condition, 13 dendritic locations tested; 25 mM BAPTA (5 cells and 6 dendritic locations tested). (Right)-Mean±SD of normalized EPSP 5–10 minutes after a conditioning protocol in the range of 0.4–1 µM *Δ[Ca^2+^]_I_*; two-sample t-test (control and BAPTA experiments): p<0.01.

The definitive confirmation that LTP requires calcium influx via VGCCs is the application of the conditioning protocol during a complete block of postsynaptic calcium transients. To this aim, we loaded PNs with 25 mM BAPTA and 100 µM Alexa-488 after filling the tip of the patch pipette with BAPTA-free solution to allow measurements of *Δ[Ca^2+^]_i_* signals before BAPTA diffusion. In the experiment of Figure 7A, we positioned two stimulation electrodes as the dendrite became visible with Fura-FF fluorescence (∼2 minutes after breaking the seal) and measured *Δ[Ca^2+^]_i_* associated with EPSP bursts every ∼30 s. The amplitude of the *Δ[Ca^2+^]_i_* signal, adjusted to be in the LTP range, was constant for ∼10–15 minutes. After the first detection of Alexa fluorescence in the dendrite (Figure 7A), we waited ∼35 minutes to allow for dendritic BAPTA equilibration and to test the conditioning protocol. No LTP was observed. The same result was obtained with two stimulating electrodes in 6 experiments (Figure 7B). Figure 7C shows the scatter plot of the EPSP change 5–10 minutes after the conditioning protocol relative to the initial value against the *Δ[Ca^2+^]_i_* signal in the presence of BAPTA, together with the data points in control conditions in the range of 0.4–1 µM *Δ[Ca^2+^]_i_*. With intracellular BAPTA, the fractional change of the EPSP amplitude was 1.00±0.09 (N = 6), smaller than that observed in control conditions (1.38±0.19, N = 13, p<0.01 two-sample t-test).

**Figure 8:**
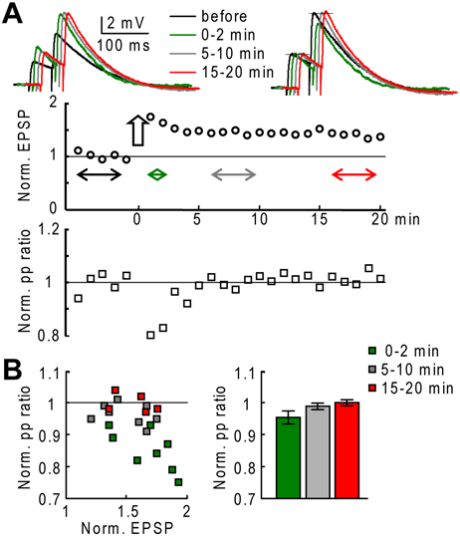
Postsynaptic expression of PF-LTP. A. (Top)-EPSP traces following paired-pulse stimulation (50 ms stimulus interval) before, 0 to 2 minutes after, 5 to 10 minutes after and 15–20 minutes after a conditioning protocol; normalized traces on the right. (Middle)-Time course of the normalized EPSP amplitudes (20 EPSPs in 5 minutes); the arrow indicates the time of the conditioning protocol. (Bottom)-Time course of the ratio between the second and the first EPSP amplitude (paired-pulse ratio) normalized to mean control value. B. (Left)-Scatter plot of the mean normalized paired-pulse ratio 0–2 minutes, 5–10 minutes and 15–20 minutes after a conditioning protocol. (Right)-Mean±SD of normalized paired-pulse ratio 0–2 minutes, 5–10 minutes and 15–20 minutes after a conditioning protocol.

To explore LTP expression, in N = 8 cells the effect of the conditioning protocol was tested on the paired-pulse ratio of 2 EPSPs at 20 Hz. In the experiment of Figure 8A, a decrease in the paired-pulse facilitation by ∼20% was observed in the first 2 minutes after the conditioning protocol, corresponding to the post-tetanic potentiation. The change in the paired-pulse facilitation was reduced to less than 5% after 5–10 minutes and after 15–20 minutes, corresponding to the LTP phase. As shown by the scatter plot of Figure 8B, this behavior was observed in all the 8 cells tested in this way. A change of the paired-pulse ratio from the control value (ratio: 0.85±0.06; p<0.05, paired t-test) was observed only in the first two minutes after the conditioning protocol.

Altogether, these experiments demonstrate that PF-LTP is induced and expressed postsynaptically.

### PF-evoked and AF-evoked dendritic firing and LTP in coronal slices

**Figure 9:**
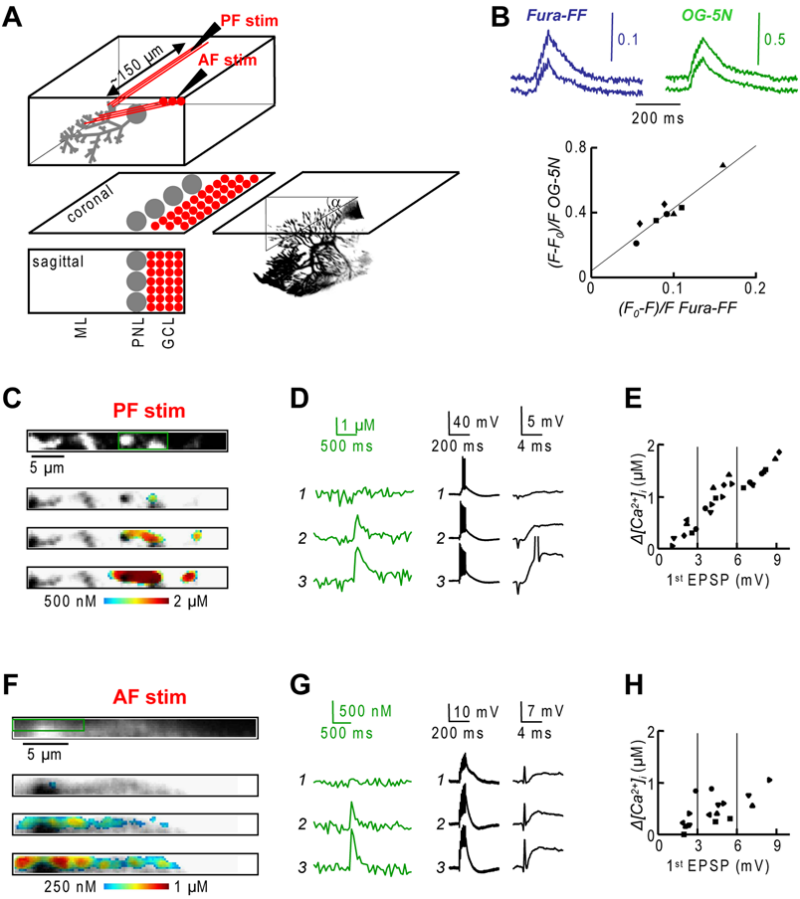
Dendritic PF and AF *Δ[Ca^2+^]_i_* signals in coronal slices. A. (Left)-Schematic of a sagittal/coronal section of the cerebellum with Molecular Layer (ML), PN Layer (PNL) and Granule Cell layer (GCL); PF stimulation: stimulation in the ML ∼150 µm from the dendritic plane; AF stimulation: stimulation in the GCL behind the PN. (Right)-3D reconstruction of a PN in a coronal slice; the angle α from the plane of the slice indicated. B. Relative calibration of fractional fluorescence changes of Fura-FF and OG-5N. (Top)-Recordings from 1 cell, 2 stimulation intensities. (Bottom)-Scatter plot from 4 cells, 2 stimulation intensities, each symbol represents a cell; linear least square fit indicated. C. Region in the coronal section of a PN dendritic tree (top) and color-coded peak *Δ[Ca^2+^]_i_* (bottom) superimposed over the fluorescence image associated with PF stimulation at three stimulation intensities. D.-*Δ[Ca^2+^]_i_* recordings from the region in the image shown in C and corresponding somatic recordings at different time scales. E.-Scatter plot of peak *Δ[Ca^2+^]_i_* following PF stimulation against the amplitude of the first EPSP; each symbol is a different cell; *Δ[Ca^2+^]_i_* signals corresponding to a first EPSP of 3–6 mV in the range of LTP induction. F, G and H. Same as C, D and E in cells where EPSPs and *Δ[Ca^2+^]_i_* signals were elicited by AF stimulation; *Δ[Ca^2+^]_i_* signals in the range of LTP induction.

It has been shown that PF synapses and synapses from cerebellar granule cells (CGCs) formed in the ascending tract [21] have different susceptibility for synaptic plasticity [22], [23]. This phenomenon was attributed to the different spatial arrangements of PF and AF afferents [24]. To discriminate PF and AF inputs, we did experiments in coronal slices as described by Sims and Hartell [22], [23] and by Marcaggi and Attwell [24]. In this preparation, the organization of synaptic inputs is preserved and the dendritic plane of PNs is perpendicular to the slice with a variable descending angle (Figure 9A). We selected neurons with dendrites positioned at an angle of ∼30°–45° from the slice plane and imaged the entire dendrite over multiple planes to localize and quantify *Δ[Ca^2+^]_i_* signals using two-photon microscopy. We used the non-ratiometric low-affinity indicator Oregon Green BAPTA-5N (OG-5N) and calibrated its fluorescence change against Fura-FF (Figure 9B) in order to compare calcium fluorescence signals.

In the first series of experiments (N = 7 cells), we positioned the stimulating electrode in the molecular layer (ML) ∼150 µm from the monitored PN to stimulate PFs. In the experiment of Figure 9C, after having localized the scanning plane with the largest dendritic *Δ[Ca^2+^]_i_* signal, we recorded calcium fluorescence over small areas evoked by EPSP bursts at different stimulation intensities. PF-EPSP bursts locally excited the dendrite leading to *Δ[Ca^2+^]_i_* signals in the micromolar range (Figure 9D). The relation between the amplitude of the first EPSP and the *Δ[Ca^2+^]_i_* signal was almost linear up to ∼9 mV and *Δ[Ca^2+^]_i_* signals of ∼2 µM in all the 7 cells tested (Figure 9E). In general, we observed that *Δ[Ca^2+^]_i_* signals that are expected to induce LTP were always associated with EPSP bursts in which the amplitude of the first EPSP was in the range of 3–6 mV.

In the second series of experiments (N = 6 cells), we positioned the stimulating electrode in the granule cell layer (GCL) behind the monitored PN allowing for AF stimulation. In these experiments, particular care was observed to keep the stimulation intensity below the threshold for stimulating the CF. In the experiment of Figure 9F, the AF-EPSP bursts, albeit sparser, could still evoke dendritic spikes (Figure 9G). In all the cells, compared to the PF stimulation, *Δ[Ca^2+^]_i_* signals were smaller, not linearly related with the first EPSP amplitude (Figure 9H) and generally less localized. These signals, however, could still reach values compatible with LTP induction.

**Figure 10:**
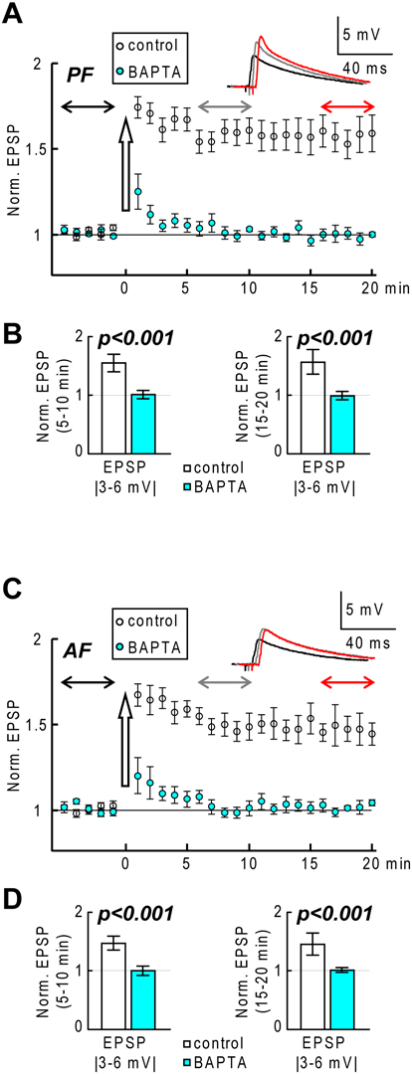
PF-LTP and AF-LTP in coronal slices. A. (Top)-Averaged EPSP traces following PF stimulation in coronal slices before, 5 to 10 minutes after and 15–20 minutes after a conditioning protocol. (Bottom)-Time course over 6 cells (Mean±SD) of the normalized EPSP in the range of 3–6 mV before and after a conditioning protocol; white symbols: control internal solution; colored symbols: 25 mM BAPTA. B. Mean±SD over 20 EPSPs evoked 5–10 minutes and 15–20 minutes after a conditioning protocol; experiments with control solution (N = 6 cells) and with 25 mM intracellular BAPTA in the pipette (N = 6 cells); two-sample t-test (control and BAPTA experiments): p<0.001. C and D. Same as A and B in cells where EPSPs were elicited by AF stimulation; experiments with control internal solution (N = 6 cells) and with 25 mM intracellular BAPTA (N = 6 cells); two-sample t-test (control and BAPTA experiments): p<0.001.

In the final set of experiments–which did not include calcium measurements-we tested the conditioning protocol, applied either to the PF or to the AF pathways in coronal slices. We adjusted the stimulation intensity to obtain the first EPSP amplitude of 3–6 mV, leading to *Δ[Ca^2+^]_i_* signals in the expected PF-LTP range. For PF stimulation (N = 6 cells), the conditioning protocol induced LTP (fractional change of EPSP amplitude after 15–20 minutes: 1.56±0.21), whereas no change in the PF-EPSP amplitude was observed in another set of 6 neurons filled with 25 mM BAPTA (fractional change of EPSP amplitude after 15–20 minutes: 0.99±0.07) as shown in Figure 10A and Figure 10B. We repeated the same test for AF-EPSPs (N = 6 cells in control internal and N = 6 cells with 25 mM BAPTA) using the same EPSP amplitude. LTP was also observed for AF-EPSPs (fractional change of EPSP amplitude after 10–15 minutes: 1.45±0.19), but not in the cells filled with BAPTA (fractional change of EPSP amplitude after 10–15 minutes: 1.01±0.04) as shown in Figure 10C and Figure 10C.

In summary, dendritic calcium spikes and associated LTP were detected following both PF and AF stimulation.

## Discussion

In this report, we describe three novel findings that significantly further our understanding of the dendritic mechanisms underlying PN synaptic plasticity. First, we show that local high-frequency dendritic spikes generate *Δ[Ca^2+^]_i_* signals that summate non-linearly because they transiently saturate the ECB. Second, we demonstrate that dendritic calcium spikes are associated with the induction of postsynaptic PF-LTP. Third, we report that dendritic calcium firing leading to LTP can occur not only by activation of adjacent PF-EPSPs, but also by activity in the sparser AF tract, implying a less stringent spatial organization of synaptic inputs compared to the one necessary for mGluR1- and endocannabinoid-mediated PF-LTD [25].

### Dendritic calcium spikes can saturate the endogenous calcium buffer

The ability of a cell to dynamically regulate calcium depends on the kinetic properties of the calcium-binding proteins forming the ECB as well as on the time course of the calcium signal [26]. When more spikes occur sequentially, *Δ[Ca^2+^]_i_* signals summate non-linearly if the associated calcium influx partially saturates calcium-binding molecules. Transient ECB saturation following action potentials has been shown to occur presynaptically and to contribute to short-term plasticity [27]. In cultured PNs, pulses of somatic depolarization have been reported to progressively saturate the fast ECB [19]. Here, we show that local dendritic calcium spikes can transiently saturate the ECB leading to long-term synaptic plasticity. The evidence that the amplitude of the *Δ[Ca^2+^]_i_* signal depends on the size of the activated dendritic region suggests that the saturated ECB involves mostly mobile molecules re-equilibrating over relatively small volumes. The PN is characterized by an exceptionally large equilibrium buffering capacity estimated at ∼2000 [28]. Both slow calcium-binding proteins like parvalbumin [18] and fast-binding calcium-binding protein like calbindin D_28k_ [17] contribute to the equilibrium ECB of PNs [29]. Computer simulations presented here show that the supra-linear *Δ[Ca^2+^]_i_* summation is due to saturation of fast-binding ECBs such as calbindin D_28k_ or other molecules, as already suggested in another study [19]. It is important to note that our experimental approach, utilizing combined voltage and calcium imaging, allowed to observe that the supra-linear summation of *Δ[Ca^2+^]_i_* signals is independent of the increase in calcium influx per spike providing, for the first time, a direct demonstration for ECB saturation associated with dendritic depolarization.

### Dendritic calcium spikes can induce long-term plasticity

In this report, we observed that several consecutive dendritic spikes at 100 Hz can induce PF-LTP. Postsynaptic PF-LTP is necessary as a reversal mechanism for PF-LTD and its physiological occurrence is supported by *in vivo* receptive field plasticity [30]. The induction mechanism used here is novel, because our conditioning protocol differs from those previously described [20], [31], but the calcium dependence of the polarity of plasticity confirms what was already reported [20]. In our study, however, we could correlate the occurrence of calcium spikes with the induction of LTP without activation of NMDA or mGluR1 receptors. The induction of LTP by only calcium increase is still a controversial issue [32]. The present study cannot exclude the involvement of signaling within PF synaptic transmission.

Repetitive bursts of PF-EPSPs followed by 1 or 2 CF-EPSPs can induce PF-LTD [4], [33] mediated by mGluR1 activation. The underlying pairing protocol occurs with a concomitant supra-linear calcium signal [4] associated with the CF-EPSP. However, as reported by Brenowitz and Regehr [12], not only is the dendritic component of this supra-linear calcium signal independent of mGluR1 activation and calcium release from stores, but the delay between PF and CF stimulation differs from that responsible for the mGluR1- and endocannabinoid-mediated short-term plasticity. Here we demonstrate that the CF-associated supra-linear *Δ[Ca^2+^]_i_* signal is also due to local ECB saturation.

Data reported in the Supporting Information file Data S1 indicates that the priming calcium signal, necessary for InsP_3_-mediated calcium release from stores [34] and generally provided by the CF-evoked spike [35], can be replaced by PF-evoked dendritic spikes. If this is the case, what is the mechanism of PF-CF coincident detection? We don’t have an answer to this question, but the scenario appears to be a dynamic puzzle involving differences in calcium signaling [30] and other signaling aspects so far not investigated.

### Calcium spikes decode the architecture of presynaptic fibers

CGC axons ascend from their original layer and bifurcate perpendicularly to form beams of parallel trajectories extending for several millimeters. Synapses to PNs are formed both in the ascending tract [21] and through the parallel trajectories over long distances from the branching point [36]. Different biophysical mechanisms can interplay to decode the arrangement of presynaptic fibers into postsynaptic signaling. A first mechanism is glutamate spillover that occurs only in adjacent synapses and regulates mGluR1 activation and LTD [25]. Here we show that the dendritic calcium burst is another mechanism that can decode the architecture of presynaptic fibers into synaptic plasticity. This property relies on the ability of PF/AF synapses to facilitate and elicit highly-localized calcium bursts, in contrast to the widespread CF-mediated dendritic spike.

In contrast to the mGluR1- and endocannabinoid-mediated LTD and to other forms of synaptic plasticity, that are exclusive of PF synapses [22], [23], [24] the LTP described here can also be induced by the sparser AF activation. Nevertheless, a difference in susceptibility for this type of plasticity cannot be excluded from the present experiments.

In summary, glutamate spillover necessary for the activation of mGluR1 [25] and dendritic calcium bursts can decode the spatial organization of CGC-PN synapses at two different levels. The fine structure of PF-adjacent synapses can be decoded by the mGluR1 activation and by the local release of endocannabinoids leading to LTD. The gross structure of PF/AF synapses can be decoded by the local depolarization above the threshold for calcium firing leading to LTP.

## Materials and Methods

### Slice preparation and electrophysiology

Experiments, approved by Basel cantonal authorities, were done in 250 µm thick sagittal or in 300 µm thick coronal cerebellar slices from 25–35 days old mice (C57BL/6, body weight 10–19 g), decapitated following isoflurane anaesthesia (according to the Swiss regulation). Slices were prepared in ice-cold solution using a HM 650 V vibroslicer (Microm, Germany), incubated at 35°C for 40 minutes and maintained at room temperature. Somatic whole-cell recordings were made at 32–34°C using a Multiclamp 700A amplifier (Axon Instruments, USA) under an upright microscope (Olympus BX51-WI). The extracellular solution contained (in mM): 125 NaCl, 26 NaHCO_3_, 20 glucose, 3 KCl, 1 NaH_2_PO_4_, 2 CaCl_2_ and 1 MgCl_2_, pH 7.4 when bubbled with a gas mixture containing 95% O_2_, 5% CO_2_. The basic intracellullar solution contained (mM): 120 KMeSO_4_, 10 NaCl, 4 Mg-ATP, 0.3 Tris-GTP, 14 Tris-Phosphocreatine, 20 HEPES (pH 7.3, adjusted with KOH) and indicators were added to the internal solution. In experiments with either JPW-1114 or the combination BAPTA+Alexa-488, electrodes were front-filled with just the basic internal solution. In the case of BAPTA+Alexa-488, the amount of front-filled solution was adjusted to delay the diffusion of the back-filled solution into the tip of the pipette by ∼10 minutes. Local stimulation of presynaptic fibers was carried out with patch pipettes filled with extracellular solution positioned using hydraulic manipulators (Narishige, Japan). Somatic electrical signals were filtered at 4 kHz and acquired at 8 kHz or at 16 kHz using the A/D board of the Redshirt imaging system.

### Optical recordings

Experiments on sagittal slices were carried out by exciting fluorescence with a 150 W xenon lamp (CAIRN Research Ltd., Faversham, UK) and by imaging with a 80×80 pixels CCD camera NeuroCCD-SM (RedShirtImaging LLC, Decatur, GA, USA). The excitation light was directed to a water immersion objective Olympus 60X/1.1 NA and the fluorescent image of the cell projected via a 0.25X optical coupler onto the CCD camera. The imaged field was ∼125 µm×125 µm (80×80 pixels). The excitation light was directed either to a filter cube for the voltage imaging (excitation: 525±25 nm; dichroic mirror >570 nm; emission filter >610 nm) or to another cube for calcium imaging (excitation 387±6 nm; dichroic mirror >470 nm; emission 510±42 nm).

The procedure to achieve combined voltage and calcium recordings has been previously described [37], [5]. Voltage and calcium fluorescence were sampled at 2000 frames/s and 500 frames/s respectively. Fractional changes of fluorescence were converted into *ΔV_m_* using prolonged hyperpolarizing pulses as described in another report [5]. We estimated *Δ[Ca^2+^]_i_* from the equation:
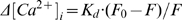
(1)where *F* is the fluorescence after auto-fluorescence subtraction and the fluorescence at 0 and saturating Ca^2+^ were approximated with the initial fluorescence (*F_0_*) and with the auto-fluorescence respectively. We used *K_d_* = 10 µM for Fura-FF [14]. In this condition, the dye buffering capacity was negligible (only ∼5%) compared to the estimated equilibrium ECB of the PN [25].

For BF2 experiments, we estimated the amount of calcium bound to the non-saturated high-affinity calcium indicator BF2, (i.e. the integral of the calcium influx), using the fractional change of BF2 fluorescence:
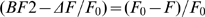
(2)


Two-photon measurements of *Δ[Ca^2+^]_i_* signals in coronal slices were done with a tunable, mode-locked titan sapphire laser (MaiTai HP, Spectra Physics Germany) set to 800 nm and a confocal laser scanning system (FV300, Olympus Switzerland) and a high-aperture 20x water- immersion lens (Olympus LUMPLAN 20x) by scanning multiple sections to localize the dendritic site where the largest calcium signal was observed. Areas of typically 80–150 by 8 pixels were scanned at 17–25 Hz to measure local dendritic signals with minimal under-estimate of the signal amplitude and distortion of its spatial distribution.

### Data analysis

Images and electrophysiological recordings were analyzed with dedicated software written in Matlab (The MathWorks Inc., Natick, MA, USA). To compare voltage and calcium measurements, we routinely checked individual trials for consistency and averaged several recordings to improve the signal-to-noise ratio. All optical voltage or calcium traces reported in the figures are averages of 4–9 recordings.

The amplitude of synaptic responses was tested by evoking individual EPSPs every 15 s (4 EPSPs every minute). We routinely recorded EPSPs for 5 minutes (20 recordings) before a conditioning protocol and for 10–20 minutes after the conditioning protocol. For the analysis, 4 EPSPs (1 minute) or 20 EPSPs (5 minutes) were averaged and the peak EPSP amplitude normalized to that of the 20 averaged EPSPs before the conditioning protocol.

Conditioning protocols with peak mean *Δ[Ca^2+^]_i_* signals >2 µM were associated with EPSP bursts with the first EPSP generally >8 mV that occasionally fired somatic action potentials. In those cases, the estimate of the effect of the conditioning protocol, on the EPSP evoked with the same intensity, was unreliable because the occasional action potentials prevented the measurement of the peak EPSP amplitude. Therefore, for the EPSP test before and after the conditioning protocol, the stimulation was set to evoke an EPSP of ∼6 mV, using an intensity value lower than that used for the conditioning protocol.

Results from two-sample or paired t-tests were considered significantly different for p<0.01 and not significantly different for p>0.1. In individual experiments, a conditioning protocol was defined to induce LTP or LTD when the p value of the two-sample t-test (N = 20 samples) on the EPSP amplitudes before and after the conditioning protocol was <0.01.

### Computer simulations

Computer simulations of free calcium concentration (*[Ca^2+^]*) dynamics were done using the modified model described by Schmidt et al [13], with a single cylinder compartment with 10 µm length and 1 µm radius (surface *A* and volume *V*). The variables included the concentrations of the free calcium indicator Fura-FF (*[FF]*), of the free ECBs calbindin D28k *([CB]*) and parvalbumin (*[PB]*), of the three buffers bound to calcium (*[Ca^2+^FF]*, *[Ca^2+^CB]* and *[Ca^2+^PV]*), and of the parvalbumin bound to magnesium (*[Mg^2+^FF]*). We took into account the 4 binding sites of CB with faster and slower kinetics (ratio 2:2) and the 2 binding sites of PV [13]. The constant magnesium concentration *[Mg^2+^]* = 620 µM was calculated from 4 mM Mg-ATP in our internal solution using WinMaxC (http://www.stanford.edu/~cpatton/maxc.html). The model also incorporated a Michaelis-Menten extrusion mechanism
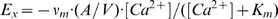
(3)with maximal pump velocity *ν_m_* = 300 pM·cm^−2^·s^−1^ and Michaelis-Menten constant *K_m_* = 3 µM and a leak current
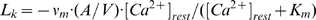
(4)necessary to balance the clearance of calcium at its resting value *[Ca^2+^]_rest_* = 45 nM [13]. Calcium influx (*I_n_*) associated with calcium spikes was calculated from the calcium current *I*, approximated with windows of 100 pA amplitude and 3 ms duration, using the expression
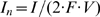
(5)where F is the Faraday’s constant. The change of free calcium concentration in a time step *Δt* (1 µs in our simulations) due to the binding of calcium to the buffer binding site BS is given by
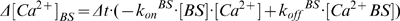
(6)where [BS] is the “concentration” of the binding site and *k_on_^BS^* and *k_off_^BS^* are the on- and off- rates of BS for calcium.

The total change in free calcium concentration is therefore given by:
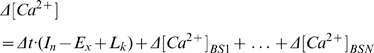
(7)where *N* is the total number of binding sites. The change in concentration of BS bound to calcium is given by
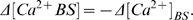
(8)


To account for the interaction between PV and magnesium, the change in free PV is given by:
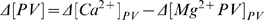
(9)where the change in concentration of BS bound to magnesium is
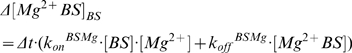
(10)where *k_on_^BSMg^* and *k_off_^BSMg^* are the on- and off- rates of BS for magnesium. For CB and FF, the change in free concentration is
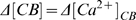
(11)

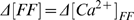
(12)


For the kinetics of CB and PV binding to calcium, we used the parameters reported by Nägerl et al. [17] and Lee et al. [18] and doubled the on-rate (and the affinity) to account for the temperature increase of ∼10°C (*k_on_^CBfast^* = 17.0·10^7^ M^−1^·s^−1^; *k_off_^CBfast^* = 35.8 s^−1^; *k_on_^CBslow^* = 2.6·10^7^ M^−1^·s^−1^; *k_off_^CBslow^* = 2.6 s^−1^; *k_on_^PV^* = 2·10^7^ M^−1^·s^−1^; *k_on_^PV^* = .0.95s^−1^). For the kinetics of PV binding to magnesium, we used the parameters reported by Eberhard and Erne ([38]) (*k_on_^PVMg^* = 1.6·10^7^ M^−1^·s^−1^; *k_on_^PVMg^* = 25 s^−1^). For PV concentration, we used the value of 40 µM used by Schmidt et al. ([13]). For CB concentration, we used the value of 100 µM which is between what used by Schmidt et al. ([13]) and what reported by Maeda et al. ([19]). Finally, for FF, we set the concentration to 800 µM, *K_on_* = 5·10^8^ M^−1^·s^−1^ as used by Xu-Friedman and Regehr ([39]) and *K_d_* = 10 µM (*K_d_* = *K_off_*/*K_on_*). The set of parameters reported above were used for the first simulation described in the Results. In the analysis of ECB saturation, we aimed at establishing a role for faster ECB that were not taken into account in the model. To this purpose, in the second simulation described in the Results, we quadrupled the value of faster on-rate of CB (*k_on_^CBfast^* = 68.2·10^7^ M^−1^·s^−1^).

## Supporting Information

Data S1 Data not directly related to the main results of the report, but supporting the principal experiements
(0.41 MB DOC)
