# Supplementary material for: Dendritic Spike Saturation of Endogenous Calcium Buffer and Induction of Postsynaptic Cerebellar LTP
Source: PLoS One. 2008 Dec 23;3(12):e4011. doi: 10.1371/journal.pone.0004011 (PMC2603473; doi:10.1371/journal.pone.0004011)
Supplement: Data S1 — Data not directly related to the main results of the report, but supporting the principal experiements (0.41 MB DOC) [file pone.0004011.s001.doc]

**SUPPORTING INFORMATION**

**Marco Canepari and Kaspar E. Vogt**

***Δ[Ca^2+^]_i_* signals during a conditioning protocol**

Peak *Δ[Ca^2+^]_i_* signals during the conditioning protocol were calculated from the averaged *Δ[Ca^2+^]_i_* recording (30 trials) over the entire period of 1 minute using always the maximal 8X8 pixels region to standardize the analysis. However *Δ[Ca^2+^]_i_* signals recorded every 2 EPSP bursts were further analyzed to estimate their time course during the conditioning protocol. We looked at *Δ[Ca^2+^]_i_* of ~1 µM, inducing LTP (see example of Figure S1A) and at *Δ[Ca^2+^]_i_* >2 µM inducing LTD (see example of Figure S1B). As shown by these two examples and by Figure S1C, *Δ[Ca^2+^]_i_* signals associated with LTP do not substantially vary during the conditioning protocol whereas those associated with LTD decrease over time. The evidence that *Δ[Ca^2+^]_i_* signals in the range of 0.5-1.2 µM are stable allowed to correlate conditioning protocols where direct *Δ[Ca^2+^]_i_* measurements were not possible (see experiments of Figure 7 and of Figure 10 in the paper) with indirect estimates done either previously (experiments with BAPTA) or from other cells (experiments in coronal slices).

***Δ[Ca^2+^]_i_* mediated by mGluR1s**

Calcium release from stores via InsP_3_ receptors is primed by previous calcium signals. Although this signal primarily originates in spines, in a minority of experiments, following a PF-EPSP burst, we could clearly distinguish a *Δ[Ca^2+^]_i_* signal component with the kinetics consistent with calcium release from stores that was blocked by CPCCOEt (100 µM) or by CPA (30 µM) as shown in the two examples of Figure S1D. This evidence indicates that the calcium-priming signal mediated by the CF-EPSP can be replaced by the local dendritic firing as it can be expected from the size and from the timing of those signals. This result suggests that the PF-CF coincidence mechanism cannot be only explained in terms of different *Δ[Ca^2+^]_i_* signals.

**
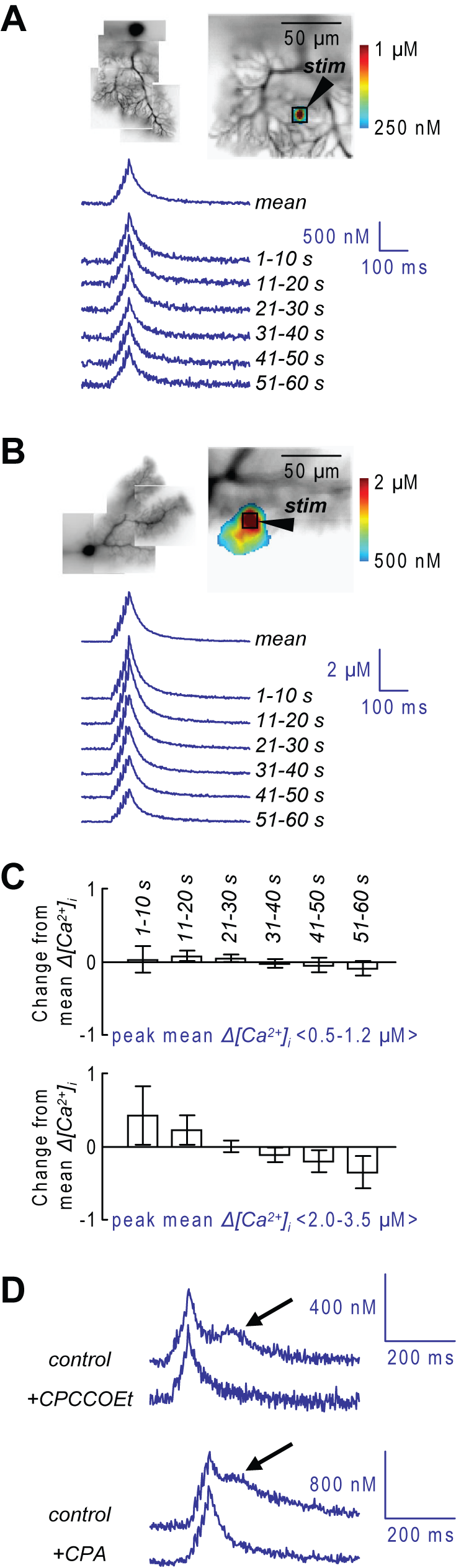
**

**Figure S1** *Δ[Ca^2+^]_i_* associated with PF-EPSP bursts. **A** (Top) –PN reconstruction and recorded dendrites and the region of maximal *Δ[Ca^2+^]_i_*. (Bottom) - Mean *Δ[Ca^2+^]_i_* during conditioning protocol (average of 30 trials over one minute) and averaged *Δ[Ca^2+^]_i_* every 10 seconds (5 trials) during conditioning protocol; mean peak *Δ[Ca^2+^]_i_* ~800 nM inducing LTP; small *Δ[Ca^2+^]_i_* fluctuations during conditioning protocol. **B** Same as a but for *Δ[Ca^2+^]_i_* mean peak > 2 µM inducing LTD; decrease of *Δ[Ca^2+^]_i_* during conditioning protocol. **C** Mean ± SD of the fractional difference of the peak mean *Δ[Ca^2+^]_i_* every 10 s relative to the peak mean *Δ[Ca^2+^]_i_* over 60 s in the 0.5-1.2 µM range (12 cells, top graph) and in the 2-3.5 µM range (7 cells, bottom graph). **D** Two examples of *Δ[Ca^2+^]_i_* signals following a PF-EPSP burst with a late mGluR1-mediated component blocked by 100 µM CPCCOEt (top) or by 30 µM CPA (bottom).
